# The Novel Elemene Derivative, OMe-Ph-Elemene, Attenuates Oxidative Phosphorylation and Facilitates Apoptosis by Inducing Intracellular Reactive Oxygen Species

**DOI:** 10.3390/antiox13121499

**Published:** 2024-12-09

**Authors:** Jianhua Guo, Jiayi Wang, Shuhao Fan, Mucong Gao, Guodu Liu, Yong Xia

**Affiliations:** 1 Cheeloo College of Medicine, Shandong University, Jinan 250012, China; 202217849@mail.sdu.edu.cn; 2 Key Laboratory for Chronic Non-Communicable Diseases of Shandong Province, Institute of Precision Medicine, College of Medical Engineering, Jining Medical University, Jining 272067, China; wangjiayi@stu.mail.jnmc.edu.cn (J.W.); fanshuhao55@gmail.com (S.F.); gaomucong@stu.mail.jnmc.edu.cn (M.G.); 3 Inner Mongolia Key Laboratory of Fine Organic Synthesis, College of Chemistry and Chemical Engineering, Inner Mongolia University (South Campus), Hohhot 010030, China

**Keywords:** Elemene, colorectal cancer, oxidative stress, oxidative phosphorylation, apoptosis

## Abstract

The incidence and mortality rates of colorectal cancer have been steadily increasing, making it one of the most prevalent cancers globally. Although current chemotherapy drugs have shown some efficacy in treating this disease, their associated side effects necessitate the development of more effective treatments and medications. The clinical application of elemene is widely utilized in tumor treatment; however, its efficacy is hindered by the requirement for high dosage and suboptimal anticancer effects. Thus, we have made modifications and enhancements to elemene, resulting in the development of a novel compound named (E)-8-(3,4,5-OMe-Ph)-β-Elemene (abbreviated as OMe-Ph-Elemene) that demonstrates significantly enhanced efficacy in suppressing colorectal cancer. We conducted an in vivo study and demonstrated the potential of OMe-Ph-Elemene in suppressing the growth of colorectal cancer xenograft tumors in zebrafish. The in vitro experiments revealed that OMe-Ph-Elemene effectively inhibited the proliferation and migration of colorectal cancer SW480 and HT-29 cells by inducing reactive oxygen species (ROS)-caused apoptosis and inhibiting mitochondrial oxidative phosphorylation. The mechanism was elucidated through high-throughput proteomic analysis and molecular biological analysis, revealing that OMe-Ph-Elemene induced cellular oxidative stress by downregulating CISD3 and promoted cell apoptosis by downregulating TRIAP1 and upregulating HMOX1. Furthermore, OMe-Ph-Elemene suppressed colorectal cancer cells’ mitochondrial oxidative phosphorylation by downregulating NDUFA7. In summary, the utilization of the elemene parent nucleus structure has led to the derivation of a novel tumor suppressor compound characterized by high efficacy and low toxicity, thereby providing a significant reference for the development of innovative drugs for colorectal cancer treatment.

## 1. Introduction

Colorectal cancer ranks as the third leading cause of global cancer mortality, with over 1.85 million cases and 850,000 deaths annually [1]. Recent studies have indicated that the increasing incidence of colorectal cancer among individuals under 50 is attributed to obesity and unhealthy dietary habits [2]. Presently, standard treatment for colorectal cancer involves surgery, chemotherapy, and radiation therapy; however, these treatments are associated with numerous side effects due to their non-specificity and cytotoxicity towards all cells. Many patients experience recurrence following a course of treatment. Therefore, it is imperative to conduct research on more effective treatments and therapeutic drugs for colorectal cancer [3].

Recent studies have demonstrated that natural compounds can elicit anti-cancer effects through a diverse array of pharmacological mechanisms, encompassing anti-proliferation, anti-angiogenesis, anti-migration, anti-invasion, induction of cell cycle arrest, and facilitation of tumor cell apoptosis [4,5].

Turmeric, a plant belonging to the genus Curcuma, is a traditional Chinese herb that has been utilized for centuries as a hemostatic agent [6]. The compound elemene, derived from its rhizome, has emerged as a promising candidate for anti-cancer therapeutics [7,8]. The anticancer properties of elemene against liver cancer, gastric cancer, colorectal cancer, and brain cancer have been extensively demonstrated in numerous studies [9,10]. The clinical use of preparations containing elemene in combination with chemotherapy has been approved for the treatment of cancer In China [11,12]. However, due to the high effective concentration required, colorectal cancer cells exhibit reduced sensitivity to elemene. Leveraging this characteristic, researchers led by Liu Guodou at Inner Mongolia University have modified the chemical structure of elemene while preserving its core nucleus to produce a series of derivatives. The present study demonstrates that oMe-Ph-Elemene effectively preserves the low toxicity profile of elemene while significantly enhancing its efficacy in sensitizing colorectal cancer cells. As a result, it exerts potent anticancer effects on colorectal cancer cells with a much lower IC_50_ value.

Through chemical modification of elemene, OMe-Ph-Elemene has demonstrated enhanced anti-cancer activity against colorectal cancer compared to elemene. However, the mechanism by which OMe-Ph-Elemene inhibits colorectal cancer remains unclear. To investigate the underlying mechanism, we conducted experiments and found that OMe-Ph-Elemene effectively suppressed the proliferation, migration, and clonogenic ability of SW480 and HT-29 cells, as evidenced by the cell counting kit-8 (CCK-8), clonogenic assay, cell migration assay, and 5-ethynyl-2′-deoxyuridine (EDU) assay. Additionally, proteomic analysis revealed that OMe-Ph-Elemene induced oxidative stress by downregulating CISD3 promoted cell apoptosis by downregulating TRIAP1 and upregulating HMOX1, and inhibited mitochondrial oxidative phosphorylation by downregulating NDUFA7. In vivo studies further confirmed the inhibitory effect of OMe-Ph-Elemene on tumor growth in a colorectal cancer xenograft zebrafish model. In conclusion, our findings contribute to a deeper understanding of the anti-cancer mechanism of OMe-Ph-Elemene in colorectal cancer and may provide valuable insights for drug development and treatment strategies.

## 2. Materials and Methods

### 2.1. Cell Culture

Human colorectal cancer cell lines SW480, HT-29, as well as human fetal kidney cells 239T, were purchased from the American Type Culture Collection (ATCC) (Manassas, VA, USA) and cultured in DMEM medium (C3113-0500, VivaCell, Denzlingen, Germany) supplemented with 10% Fetal Bovine Serum (2375386CP, GBICO, Grand Island, NY, USA) and 1% Penicillin-Streptomycin (P/S, UB89609, BIOODIN, Guangzhou, China). The cells were then incubated at 37 °C with 5% CO_2_ in a humidified incubator.

### 2.2. Chemicals

Elemene (HY-107324) is purchased from MCE (Monmouth Junction, NJ, USA). OMe-Ph-Elemene was provided by the research group of Liu Guodou from Inner Mongolia University, and DMSO (dimethyl sulfoxide, D8371) was purchased from Solarbio Technology Co., Ltd. (Beijing, China).

### 2.3. Cell Viability Assay

The cells were inoculated in a 96-well plate at an initial confluence density of 25%. When the cell density reached approximately 40%, they were treated with elemene (0–500 µM) and OMe-Ph-Elemene (0–120 µM) based on the cell density. The relative activity of SW480, HT-29, and 239T after treatment with elemene and OMe-Ph-Elemene was assessed using the CCK8 (CK04, DOJINDO, Mashiki, Japan) assay following the manufacturer’s instructions.

### 2.4. Cell Morphological Assay

SW480 and HT-29 cells were seeded at a density of 1 × 10^5^ cells/well in 12-well plates and allowed to adhere and recover to normalcy. The cell morphology at T0 was recorded, and then the cells were treated with OMe-Ph-Elemene at a concentration range of 0–80 µM for 48 h. The morphological changes at different time points were recorded using an inverted microscope (Nikon, Tokyo, Japan).

### 2.5. Cell Migration Assay

SW480 and HT-29 cells were seeded at a density of 2 × 10^5^ cells/well in the upper chamber of the transwell, and pure DMEM was added to the upper chamber. The lower chamber was filled with DMEM containing 20% FBS and various concentrations of OMe-Ph-Elemene. The cells were then incubated in a 37 °C, 5% CO_2_ incubator for 24 h. Subsequently, crystal violet staining was utilized to quantify the number of cells that had migrated through the filter membrane following treatment with different concentrations of OMe-Ph-Elemene.

### 2.6. Clone Formation Assay

The SW480 and HT-29 cells were plated at a density of 500 cells per well in 12-well plates and incubated for 48 h. Subsequently, varying concentrations of OMe-Ph-Elemene were introduced, and the culture medium containing OMe-Ph-Elemene was refreshed every three days. The cells were then subjected to crystal violet staining solution (KGA229, KeyGEN BioTECH, Nanjing, China) for 5–10 min under a microscope (Nikon, Tokyo, Japan) to observe changes in colony formation.

### 2.7. Cell Membrane Staining Assay

Take an appropriate amount of SW480 and HT-29 cells and seed them in a 12-well plate. Treat the cells with OMe-Ph-Elemene for 24 h, followed by washing with PBS and fixation with 4% paraformaldehyde for 15 min. Subsequently, wash the fixed cells with PBS and stain them with dual staining solution (Hoechst 33242 at a dilution of 1:1000, C1022, Beyotime (Shanghai, China); Dio at a dilution of 1:1000, C1038, Beyotime (Shanghai, China) for 20 min. Finally, wash the cells with PBS twice before capturing images under a fluorescence-inverted microscope (Nikon, Tokyo, Japan).

### 2.8. Live/Dead Cell Detection

SW480 and HT-29 cells were seeded at a density of 1 × 10^5^ cells/well in 12-well plates and incubated until reaching approximately 50% confluence. Subsequently, OMe-Ph-Elemene was introduced, and the cell viability was assessed using the Calcein AM Cell Viability Assay Kit (C2013FT, Beyotime, Shanghai, China) under a microscope (Nikon, Tokyo, Japan).

### 2.9. Measurement of DNA Synthesis Rate by EdU Method

SW480 and HT-29 cells were seeded at a density of 1 × 10^5^ cells/well in 12-well plates and incubated until reaching approximately 50% confluence. Subsequently, OMe-Ph-Elemene was added, and the cells were treated for 48 h. The DNA synthesis rate of SW480 and HT-29 cells following OMe-Ph-Elemene treatment was assessed using the EDU-594 cell proliferation assay kit (C0078S, Beyotime, Shanghai, China). The staining results were captured using an inverted fluorescence microscope, followed by quantitative analysis performed with ImageJ software (1.54g).

### 2.10. Cell Cycle Assay

The SW480 cells treated with OMe-Ph-Elemene were centrifuged and collected. Subsequently, the cells were suspended in 70% cold ethanol, fixed on ice for 30 min, centrifuged at 2000 rpm, and then the supernatant was discarded. The cells were then precipitated with a mixed solution containing PI (KGA214, KeGEN BioTECH, Nanjing, China), RNase (ST579, Beyotime, Shanghai, China), and TritonX-100 (P0096, Beijing, China). Finally, the stained cells were analyzed using flow cytometry (Beckman, Cytoflex, Brea, CA, USA).

### 2.11. Detection of ROS by H2DCFDA

The cell-permeable ROS probe H2DCFDA (HYD0940, MCE, Monmouth Junction, NJ, USA) was utilized to monitor the alterations in ROS levels in SW480 cells induced by OMe-Ph-Elemene. The SW480 cells treated with OMe-Ph-Elemene and the control group were incubated with H2DCFDA at 37 °C and 5% CO_2_ in a dark environment for 30 min. Flow cytometry (Beckman, Cytofex, Brea, CA, USA) was employed to assess the changes in ROS levels within the cells. To investigate whether ROS is involved in the inhibition of cell viability by OMe-Ph-Elemene, SW480 cells were pre-treated with NAC (ST1546, Bioneer, Shanghai, China) for 1 h prior to incubation with 60 µM OMe-Ph-Elemene for 48 h. Subsequently, cell viability was assessed using the CCK8 assay kit (Beyotime, Shanghai, China).

### 2.12. Apoptosis Detection

The SW480 and HT-29 cells treated with OMe-Ph-Elemene were digested using EDTA-free trypsin (T1350, Solarbio, Beijing, China). Subsequently, the cells were incubated with AnnexinV-FITC (C1062M, Beyotime, Shanghai, China) and PI (KGA214, KeyGEN BioTECH, Nanjing, China) for 20 min under light protection. The apoptosis rate was then measured and recorded using flow cytometry (Beckman, Cytolfex, Brea, CA, USA).

### 2.13. Mitochondrial Membrane Potential Testing

The JC-1 (C2003S, Beyotime, Shanghai, China) Enhanced Mitochondrial Membrane Potential Assay Kit is a rapid and highly sensitive method for detecting the reduction in mitochondrial membrane potential through the conversion of JC-1 fluorescence from red to green. This assay is suitable for early apoptosis detection. The JC-1 assay kit was employed in this study to evaluate alterations in mitochondrial membrane potential in SW480 cells treated with varying concentrations of OMe-Ph-Elemene, which were cultured in a 12-well plate. Fluorescence staining was performed according to the manufacturer’s instructions, and images were captured using an inverted fluorescence microscope.

### 2.14. Proteomics

In order to assess the influence of OMe-Ph-Elemene on protein expression levels, protein extraction was carried out from three control samples (CT1, CT2, CT3) as well as three samples treated with a concentration of 60 µM OMe-Ph-Elemene for a duration of 24 h (LT1, LT2, LT3), utilizing SDT methodology provided by Applied Protein Technology located in Shanghai, China. Each sample consisted of a total cell count amounting to 1.5 × 10^7^ cells. Subsequent proteomic analysis and sequencing data conversion tasks were conducted by Protein Technology (Shanghai, China).

### 2.15. Western Blotting

The SW480 cells treated with OMe-Ph-Elemene were lysed, and protein was extracted using lysate (P0013, Beijing, China). Subsequently, the protein extract underwent SDS-polyacrylamide gel electrophoresis and was transferred onto a PVDF membrane for Western blotting. The primary antibodies used were HMOX1 (Rabbit, 1:3000, 10701-1-AP, proteintech, China), NDUFA7 (Rabbit, 1:500, 15300-1-AP, proteintech, Wuhan, China), and β-Actin (Rabbit, 1:2000, GB11001, Servicebio, Wuhan, China). Following incubation and washing steps, the immunoreactive signal was detected using an HRP-labeled secondary antibody. The secondary antibody employed was HRP sheep anti-Rabbit IgG (1:3000, AS014, ABclonal, Wuhan, China), followed by development with an ECL hypersensitive developer (P0018AS, Beijing, China).

### 2.16. In Vivo Experiment Using Zebrafish Transplantation Model

Select well-developed 48-h post-fertilization (hpf) zebrafish and SW480 cells that have been passaged for 24 h. Upon reaching 24 hpf, dechorionate the zebrafish embryos. Prepare a diluted cell fluorescent stain solution using PBS without calcium with a concentration of 5 microliters of stain per milliliter of solution. Incubate the stained cells in an incubator for 1–1.5 h followed by a brief refrigeration at 4 °C for 5–10 min. For cell injection, immobilize the zebrafish in agarose gel molds, administer anesthesia, and inject the cells into the yolk sac while maintaining consistent injection pressure and volume. Allow the injected zebrafish to recover for 4 h before selecting specimens with similar fluorescence intensity for drug exposure experiments under a fluorescence microscope. Place zebrafish individually in separate wells of a 24-well plate, ensuring that each group, including the SW480 control and OMe-Ph-Elemene drug treatment groups (at concentrations of 20, 40, and 80 micromoles), consists of more than seven fish. Then, expose them until they reach 96 hpf. Replace drugs every day during the exposure period. Following completion of drug exposure experiments, observe SW480 cell migration to the zebrafish intestine using confocal microscopy and perform statistical analysis. The zebrafish experiments were conducted following the approval of the Animal Ethics Committee at Jining Medical University, with an assigned animal ethics number of JNMC-2024-DW-231.

### 2.17. Statistics

A paired *t*-test was used to evaluate the differences between the two groups, and a one-way ANOVA was used for three or more groups, with a *p*-value of less than 0.05 considered statistically significant.

## 3. Results

### 3.1. The Inhibitory Effect of OMe-Ph-Elemene Was More Potent Compared to That of Elemene

**Figure 1:**
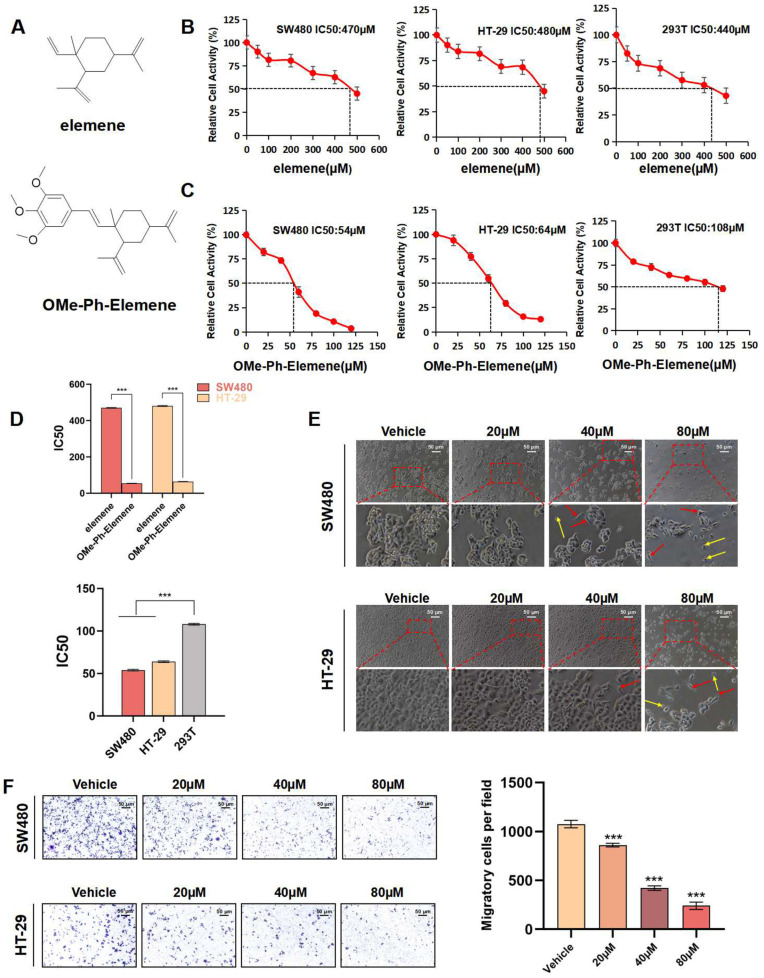
The inhibitory effect of OMe-Ph-Elemene on CRC cells was more potent compared to that of elemene. (**A**) The chemical structural formula of elemene and OMe-Ph-Elemene. (**B**) The SW480, HT-29, and 293T cells were subjected to treatment with elemene at concentrations ranging from 0 to 500 µM for a duration of 48 h. The relative cell viability was assessed using the cck8 kit, with each experimental group being replicated three times. (**C**) The SW480, HT-29, and 293T cells were exposed to OMe-Ph-Elemene at concentrations ranging from 0 to 120 µM for a duration of 48 h. The relative cell viability was assessed using the CCK-8 kit, with each experimental group replicated three times. (**D**) For SW480 and HT-29 cells, significant differences between the IC_50_ of elemene and OMe-Ph-Elemene were evaluated by a two-way *t*-test, *** *p* < 0.001. For SW480, HT-29, and 293T cells, significant differences between IC_50_ of OMe-Ph-Elemene were evaluated by one-way ANOVA analysis, *** *p* < 0.001. (**E**) The SW480 and HT-29 cells were treated with OMe-Ph-Elemene for 48 h. The morphological changes of the cells were observed using an inverted fluorescence microscope. Cell elongation was indicated by a red arrow, while cell shrinkage was indicated by a yellow arrow. (**F**) The migration of SW480 and HT-29 cells was assessed using a transwell assay following treatment with OMe-Ph-Elemene. After 24 h of exposure to various concentrations of OMe-Ph-Elemene, the number of cells that migrated across the membrane was quantified using crystal violet staining and visualized under an inverted microscope. *** *p* < 0.001. The vehicle groups represent solvent groups (DMSO).

The chemical structures of elemene and OMe-Ph-Elemene are depicted in Figure 1A. According to the CCK8 results (Figure 1B–D), OMe-Ph-Elemene exhibited a more pronounced inhibitory effect on colorectal cancer compared to elemene. The IC_50_ values of OMe-Ph-Elemene in SW480 and HT-29 cells were 54 μM and 64 μM, respectively, while the toxicity towards human fetal kidney cell 293T was lower, with an IC_50_ value of 108 μM. Additionally, the morphology of SW480 and HT-29 cells changed significantly following treatment with OMe-Ph-Elemene, resulting in more wrinkled and elongated cells as the drug concentration increased (Figure 1E). Figure 1F demonstrated that OMe-Ph-Elemene dose-dependently suppressed the migration ability of SW480 and HT-29 cells. Therefore, as the concentration of OMe-Ph-Elemene increased, there was a gradual decrease in the relative activity of the cells. Additionally, the IC_50_ value of OMe-Ph-Elemene was lower than that of elemene, indicating a more pronounced inhibitory effect on colorectal cancer by OMe-Ph-Elemene.

### 3.2. OMe-Ph-Elemene Inhibited the Proliferation of Colorectal Cancer Cells by Suppressing DNA Synthesis

**Figure 2:**
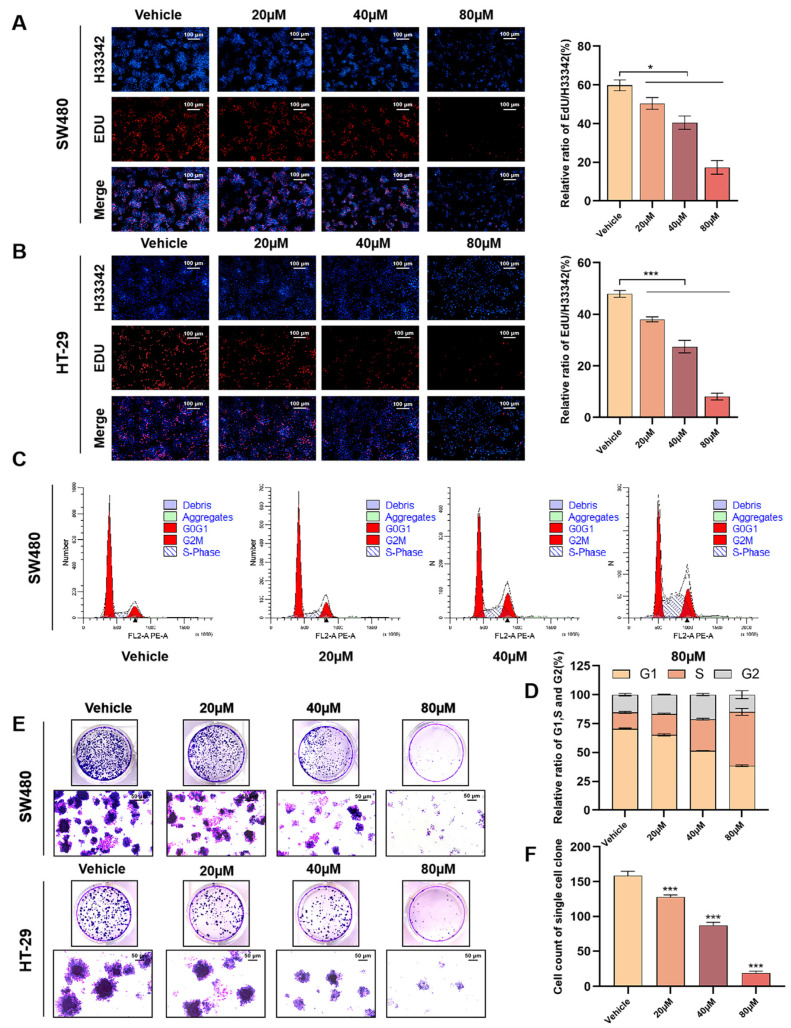
OMe-Ph-Elemene inhibited the proliferation of CRC cells by suppressing DNA synthesis. (**A**) SW480 cells treated with OMe-Ph-Elemene were stained with EDU and Hoechst 33342, and cell proliferation was observed with an inverted fluorescence microscope (EDU: red, Hoechst 33342: blue). * *p* < 0.05. (**B**) HT-29 cells treated with OMe-Ph-Elemene were stained with EDU and Hoechst 33342, and cell proliferation was observed with an inverted fluorescence microscope (EDU: red, Hoechst 33342: blue). *** *p* < 0.001. (**C**,**D**) SW480 cells treated with OMe-Ph-Elemene were stained with PI, and the cell cycle of the stained cells was detected by flow cytometry. The red peaks represented the G1 and G2 phases, and the cross regions represented the S phases. (**E**) SW480 and HT-29 cells were treated with different concentrations of OMe-Ph-Elemene for 12 days, and crystal violet-stained cell communities were observed by an inverted microscope. (**F**) A quantitative map of inhibition of OMe-Ph-Elemene on the clonal formation of SW480 cells. *** *p* < 0.001. The vehicle groups represent solvent groups (DMSO).

The rate of DNA replication serves as a crucial indicator for evaluating cell proliferation, activity, and physiological state. In this study, we observed that OMe-Ph-Elemene effectively suppressed DNA replication in SW480 and HT-29 cells through edu experiments. Upon treatment with OMe-Ph-Elemene (Figure 2A,B), there was a noticeable decrease in the total cell count. Moreover, the relative ratio of EDU/H33342 gradually declined with increasing concentrations of OMe-Ph-Elemene, indicating its potent inhibition on the DNA replication ability of SW480 and HT-29 cells. Additionally, we examined the impact of OMe-Ph-Elemene treatment on the cell cycle progression of SW480 cells. Notably, OMe-Ph-Elemene affected both G1 and S phases within the cell cycle; as its concentration increased, the G1 phase progressively decreased while the S phase gradually increased (Figure 2C,D). Furthermore, OMe-Ph-Elemene exhibited inhibitory effects on clonogenesis in both SW480 and HT-29 cells (Figure 2E,F). As the concentration of OMe-Ph-Elemene escalated, there was a notable reduction in clonoid size. These findings provide compelling evidence that by suppressing DNA synthesis, OMe-Ph-Elemene effectively impedes colorectal cancer cell proliferation.

### 3.3. OMe-Ph-Elemene Disrupted the Integrity of Cell Membranes and Induced Death

**Figure 3:**
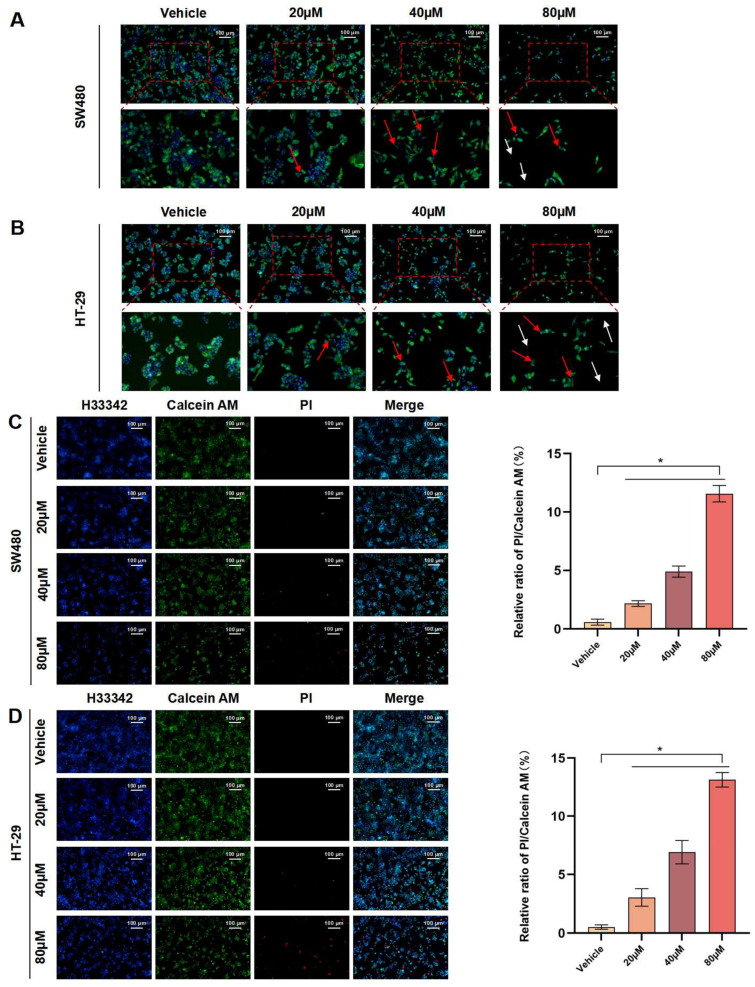
OMe-Ph-Elemene disrupted the integrity of cell membranes and induced death in CRC cells. (**A**) SW480 cells treated with OMe-Ph-Elemene were stained with Dio and Hoechst 33342, and the cell membrane showed green fluorescence, the nucleus showed blue fluorescence, the red arrow represented membrane damage, and the white arrow represented cell membrane debris. (**B**) HT-29 cells treated with OMe-Ph-Elemene were stained with Dio and Hoechst 33342, and the cell membrane showed green fluorescence, the nucleus showed blue fluorescence, the red arrow represented membrane damage, and the white arrow represented cell membrane debris. (**C**) SW480 cells treated with OMe-Ph-Elemene were stained with Hoechst 33342, Calcein AM, and PI, and the staining results were recorded by fluorescence inverted microscope (Hoechst 33342: blue, Calcein AM: green, PI: red) * *p* < 0.05. (**D**) HT-29 cells treated with OMe-Ph-Elemene were stained with Hoechst 33342, Calcein AM, and PI, and the staining results were recorded by fluorescence inverted microscope (Hoechst 33342: blue, Calcein AM: green, PI: red) * *p* < 0.05. The vehicle groups represent solvent groups (DMSO).

As the concentration of OMe-Ph-Elemene increased, there was a gradual decrease in the number of SW480 and HT-29 cells. Additionally, the cell membrane exhibited enlargement and damage, with red arrows indicating membrane damage and white arrows indicating membrane fragmentation (Figure 3A,B). In contrast, the damaging effect of OMe-Ph-Elemene on the cell membrane of normal cells, including NCM460 and 293T, is much lower compared to that observed in tumor cells (Figure S1A,B). Furthermore, in the live/dead cell staining experiment, there was a gradual decrease in the number of live cells with increasing concentrations of OMe-Ph-Elemene, while the number of dead cells showed a corresponding gradual increase (Figure 3C,D).

### 3.4. OMe-Ph-Elemene Triggered Oxidative Stress and Enhanced Cellular Apoptosis

**Figure 4:**
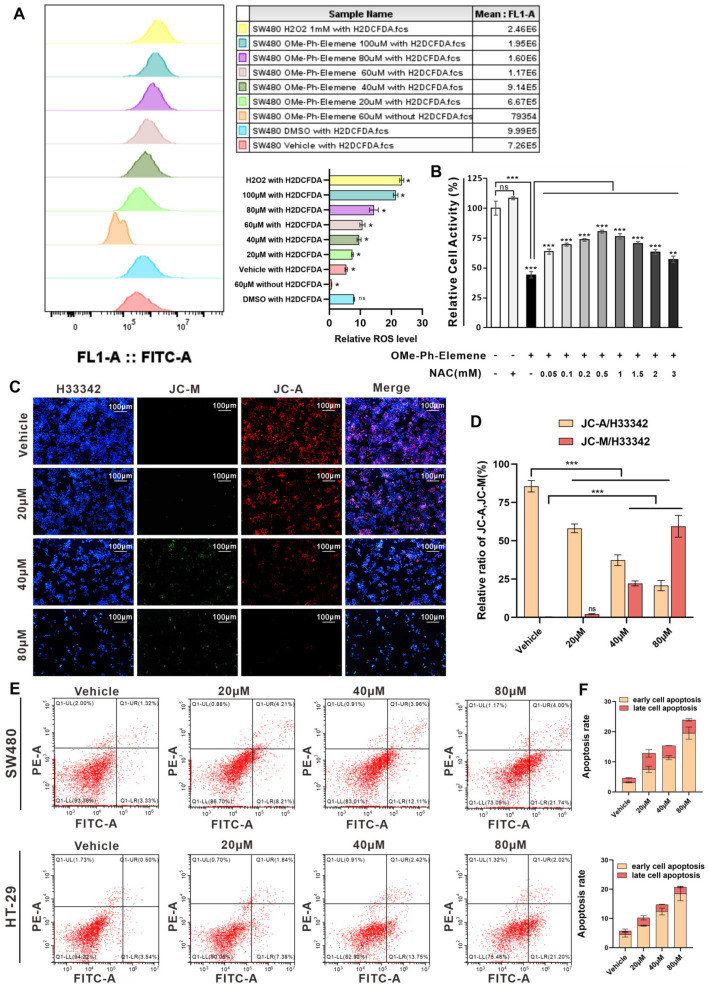
OMe-Ph-Elemene induced oxidative stress and promoted cell apoptosis. (**A**) SW480 cells treated with OMe-Ph-Elemene or H_2_O_2_ were incubated with 2µM H_2_DCFDA, and H_2_DCFDA-labeled cells were detected by flow cytometry. ns was not significant, * *p* < 0.05. The vehicle group serves as a negative control consisting solely of cells and H2DCFDA. (**B**) SW480 pretreated with 0–3 mM NAC was incubated with OMe-Ph-Elemene, and the cell activity was detected with the CCK8 kit. ns was not significant, ** *p* < 0.01, *** *p* < 0.001. (**C**,**D**) The effect of OMe-Ph-Elemene on the mitochondrial membrane potential of colorectal cancer was detected by JC-1. When the mitochondrial membrane potential is high, JC-1 forms aggregates in the mitochondrial matrix, resulting in the formation of a polymer (JC-A) that exhibits red fluorescence. Conversely, when the mitochondrial membrane potential is low, JC-1 fails to aggregate within the mitochondrial matrix and instead maintains its monomeric form (JC-M), leading to green fluorescence production. ns was not significant, *** *p* < 0.001. (**E**,**F**) SW480 and HT-29 cells treated with OMe-Ph-Elemene were stained with PI and Annexin V-FITC, and the apoptosis rate was determined by flow cytometry. The X-axis represents the FITC intensity of Annexin V on the outer membrane of the cells, while the Y-axis represents the PI staining intensity. The Q1-LR quadrant corresponds to the population of early apoptotic cells, and the Q1-UR quadrant corresponds to the population of late apoptotic cells. The vehicle groups represent solvent groups (DMSO).

In order to further investigate the anti-tumor mechanism of OMe-Ph-Elemene, we employed the H_2_DCFDA fluorescent probe to assess intracellular ROS levels following treatment with OMe-Ph-Elemene. As depicted in Figure 4A, stimulation by OMe-Ph-Elemene led to an occurrence of oxidative stress reaction, and as the concentration of OMe-Ph-Elemene treatment increased, there was a continuous elevation in intracellular ROS level. Therefore, pretreatment with the antioxidant NAC resulted in saturation of cell relative activity at a concentration of 0.5 mM NAC, and this enhanced cell relative activity could reach up to 80%, thereby indicating that OMe-Ph-Elemene induced cell death mediated by oxidative stress. The evidence supporting this claim is demonstrated by NAC preconditioning reversing oxidative stress-induced changes in SW480 cell death (Figure 4B). At the same time, MitoOSX was used to detect the changes in superoxide anion levels in mitochondria after OMe-Ph-Elemene treatment, and the results showed that OMe-Ph-Elemene could significantly up-regulate the superoxide anion in mitochondria (Figure S1C,D). A significant increase in superoxide anions led to a decrease in mitochondrial membrane potential. Furthermore, oxidative stress disrupted the integrity of the mitochondrial inner membrane and exacerbated this decline. Consequently, fluorescence staining was employed to assess mitochondrial membrane potential and revealed that OMe-Ph-Elemene reduced it in SW480 cells (Figure 4C,D). Reduction in mitochondrial membrane potential serves as an early marker for cellular apoptosis. Hence, flow cytometry was utilized for detecting apoptosis, which revealed that OMe-Ph-Elemene induced apoptosis. Moreover, as the concentration of OMe-Ph-Elemene increased, there was a gradual rise in the rate of apoptosis (Figure 4E,F). The mechanism of action of OMe-Ph-Elemene involves the induction of oxidative stress in cells, leading to a reduction in mitochondrial membrane potential and subsequent promotion of apoptosis.

### 3.5. The Zebrafish Xenograft Tumor Model and Proteomic Analysis of the OMe-Ph-Elemene Anti-Tumor Mechanism

**Figure 5:**
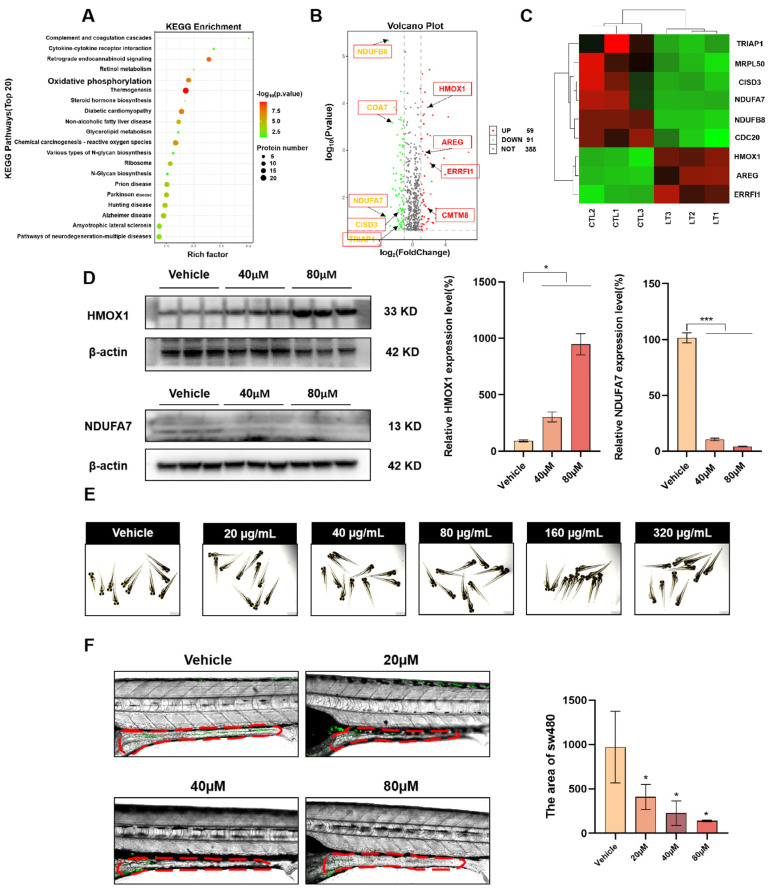
The zebrafish xenograft tumor model and proteomics results. (**A**) Proteomic KEGG enrichment analysis showed the top 20 KEGG pathways after treatment of OMe-Ph-Elemene. (**B**) The proteomic volcano map illustrates the alteration in protein levels following treatment with OMe-Ph-Elemene. The color red indicates an increase, while gold signifies a decrease. (**C**) The proteomic heat map showed that OMe-Ph-Elemene down-regulated the expression of proteins related to oxidative phosphorylation, such as NDUFA7 and NDUFB8, and up-regulated the expression of proteins related to oxidative stress and apoptosis, such as HMOX1, CISD3, and TRIAP1. (**D**) The Western blot analysis revealed a dose-dependent increase in the protein level of HMOX1, while a corresponding decrease was observed in the protein level of NDUFA7. * *p* < 0.05, *** *p* < 0.001. (**E**) The in vivo toxicity of OMe-Ph-Elemene was tested by statistical analysis of the malformation rate and mortality of zebrafish. The results showed that zebrafish below 320 µg/mL did not show deformities and death, indicating that OMe-Ph-Elemene was not significantly toxic at this concentration. (**F**) After the zebrafish transplanted tumor was treated with OMe-Ph-Elemene, the transfer of sw480 cells into the intestines of Zebrafish was observed under a confocal microscope, and the statistics were performed. * *p* < 0.05. The red dotted line is the intestinal tract of zebrafish. The vehicle groups represent solvent groups (DMSO).

To investigate the anti-cancer mechanism of OMe-Ph-Elemene, we conducted proteomic sequencing and KEGG analysis, revealing the enrichment of oxidative phosphorylation-related proteins (Figure 5A). A volcano plot in Figure 5B illustrates differentially expressed proteins between the OMe-Ph-Elemene treatment group and the control group. Following OMe-Ph-Elemene treatment, 59 proteins were upregulated while 91 proteins were downregulated. Notably, HMOX1, AREG, ERRFI1, and CMTM8 were significantly upregulated, whereas NDUFA7, NDUFB8, COA7, CISD3, and TRIAP1 were significantly downregulated. These findings are primarily associated with oxidative stress regulation and cell apoptosis, as well as oxidative phosphorylation pathways. Heat maps depicting cell apoptosis and oxidative stress markers along oxidative phosphorylation pathways show upregulation of HMOX1 and downregulation of NDUFA7 (Figure 5C). Western blot results in Figure 5D confirmed the upregulation of oxidative stress and apoptosis-related protein (HMOX1) alongside the downregulation of oxidative phosphorylation-related protein (NDUFA7). Additionally, oxygen consumption was measured to detect changes in mitochondrial oxidative phosphorylation levels of SW480 cells after OMe-Ph-Elemene treatment. The results showed that when SW480 cells were treated with 60 μM OMe-Ph-Elemene, the oxygen consumption of intracellular mitochondria and the ADP/O ratio decreased significantly, indicating that the level of oxidative phosphorylation in cells was reduced after treatment with OMe-Ph-Elemene. In addition, our proteomic GO enrichment results included many biological processes and cellular components associated with oxidative phosphorylation and electron transport chains (Figure S1E–G). We investigated the in vivo antitumor efficacy of OMe-Ph-Elemene using a zebrafish tumor transplantation model. As depicted in Figure 5E, when the concentration of OMe-Ph-Elemene reached 320 µg/mL, there was no reduction in the zebrafish population and no occurrence of malformations, indicating the low toxicity profile of OMe-Ph-Elemene. By evaluating the abundance of SW480 cells within the zebrafish gastrointestinal tract, we observed a significant reduction in comparison to the control group as the concentration of OMe-Ph-Elemene increased, suggesting that OMe-Ph-Elemene effectively suppressed the proliferation of colorectal cancer cells in vivo (Figure 5F).

## 4. Discussion

In recent years, a range of natural products derived from Chinese herbs, such as elemene and curcumin, have demonstrated their potential in inhibiting cancer through various signaling pathways, including anti-proliferation, promotion of apoptosis, and autophagy [13,14].

Elemene, a plant-derived compound with significant biological activity, has been approved as a Class II anti-tumor new drug in China [15,16]. Previous studies have shown that elemene can induce apoptosis and autophagy in colorectal cancer cells through the ROS/MAPK/mTOR pathway [9], as well as regulate the epithelial-mesenchymal transition to inhibit the invasion and metastasis of colorectal cancer cells [17,18]. However, its high effective concentration limits its efficacy in the treatment of colorectal cancer. To address this issue, we synthesized the compound OMe-Ph-Elemene through the chemical modification of elemene. The anticancer activity of OMe-Ph-Elemene extremely surpassed that of elemene, and its inhibitory effect on CRC was investigated through in vivo and in vitro experiments. Additionally, the potential molecular mechanism was explored using high-throughput proteomics.

Recent studies have revealed that tumor metastasis is often driven by a small subset of cancer cells resistant to therapeutic interventions [19]. Colorectal cancer cells not only harbor these therapy-resistant cells but also contain cancer stem cells (CSCs) with characteristics akin to normal stem cells [20,21]. Both of these cell subpopulations can be enriched following chemotherapy or radiotherapy, and while there is no consensus on the biological energy source utilized by CSCs and CRC, findings from primary malignant tumors suggest a preference for oxidative phosphorylation [22,23,24,25]. Additionally, it has been demonstrated that the expression of oxidative phosphorylation genes is inversely correlated with the survival time of cancer patients post-chemotherapy [26]. Changes in cellular energy metabolism represent a prominent characteristic of colorectal cancer. Increasing evidence suggests that there is an upregulation of oxidative phosphorylation (OXPHOS) to fulfill the energy demands associated with tumorigenesis and disease progression [27]. Our study results discovered that several mitochondrial ribosomal proteins (MRPL10, MRPL27, MRPL17, MRPL11, MRPS33, MRPS10) and oxidative phosphorylation-related proteins (NDUFA7, NDUFB8, COA7) were downregulated by OMe-Ph-Elemene. Therefore, OMe-Ph-Elemene might inhibit CRC by targeting mitochondrial proteins. The inhibition of CRC cell oxidative phosphorylation by OMe-Ph-Elemene suggests potential enhanced efficacy against cancer stem cells and therapy-resistant cells.

Studies have reported a close association between mitochondrial metabolic dysfunction and tumorigenesis [28,29,30], with tumor cells exhibiting significantly higher mitochondrial membrane potential compared to normal cells [31]. Mitochondria, the site of ROS production, plays a crucial role in cell death mechanisms [32]. Previous research has demonstrated that promoting mitochondrial ROS production can trigger apoptosis in tumor cells [33,34,35]. In this study, OMe-Ph-Elemene exerted a significant inhibitory effect on cell activity and survival by upregulating intracellular ROS levels. Moreover, OMe-Ph-Elemene notably reduced the mitochondrial membrane potential of CRC cells, and facilitated apoptosis in CRC cells, thereby confirming its potent anticancer properties. However, while it was observed that OMe-Ph-Elemene induced apoptosis in CRC cells, the possibility of other non-apoptotic modes of cell death cannot be disregarded and will be further investigated in future studies.

In our research, the treatment of colorectal cancer cells with OMe-Ph-Elemene led to the modulation of several crucial proteins associated with oxidative stress and cell apoptosis, including TRIAP1, HMOX1, and CISD3. TRIAP1 (TP53-regulated cell death inhibitor 1) has been reported to be highly expressed in various types of cancer. Kenza et al. demonstrated that the expression of TRIAP1 in cell models of colorectal cancer promotes cancer cell proliferation and tumorigenesis, while the knockdown of TRIAP1 inhibits cell proliferation and alters mitochondrial ultrastructure [36]. In this study, OMe-Ph-Elemene reduced TRIAP1 in CRC cells, leading to inhibition of colorectal cancer cell proliferation and promotion of apoptosis. HMOX1 induces hemoglobin decomposition, leading to the release of ROS and enhancing cell apoptosis under oxidative stress [37,38,39]. And the up-regulation of HMOX1 expression serves as a promoting factor for apoptosis [40]. High-throughput proteomics and WB results from this study demonstrate that OMe-Ph-Elemene significantly upregulates the expression of HMOX1. These findings suggest that the pro-apoptotic mechanism of OMe-Ph-Elemene involves the upregulation of HMOX1 to induce cell apoptosis. CISD3 is highly expressed in various human cancers, and numerous studies have demonstrated that silencing CISD3 results in heightened mitochondrial instability and iron accumulation, significantly accelerating lipid peroxidation and promoting the elevation of mitochondrial ROS [41,42]. Our findings indicate that OMe-Ph-Elemene substantially diminishes CISD3 expression in CRC cells, fosters the buildup of mitochondrial ROS, and triggers cellular oxidative stress.

This study presents the inhibitory effects of OMe-Ph-Elemene on colorectal cancer cells and its comprehensive pharmacological mechanisms. Targeting mitochondrial oxidative phosphorylation and inducing oxidative stress are two key aspects of OMe-Ph-Elemene in suppressing cancer. However, there are still limitations in this study: the direct interacting proteins of OMe-Ph-Elemene remain unidentified, and the cellular localization, as well as the effective concentration of drugs in cells, are yet to be determined. In subsequent studies, we will unveil these above scientific questions and further elucidate their molecular mechanism.

## 5. Conclusions

In summary, OMe-Ph-Elemene demonstrates efficacy as an anti-cancer compound by concentration-dependently inhibiting mitochondrial oxidative phosphorylation and promoting the accumulation of ROS. Furthermore, OMe-Ph-Elemene induces cell apoptosis through the upregulation of HMOX1. Therefore, our study has elucidated the cellular and molecular mechanisms underlying the anti-cancer effects of OMe-Ph-Elemene, providing valuable insights for the development of effective strategies for treating CRC.

## Data Availability

The mass spectrometry proteomics data have been deposited to the ProteomeXchange Consortium (https://proteomecentral.proteomexchange.org (accessed on 29 August 2024)) via the iProX partner repository with the dataset identifier PXD055327.

## Supplementary Materials

The following supporting information can be downloaded at: https://www.mdpi.com/article/10.3390/antiox13121499/s1. Figure S1: OMe-Ph-Elemene induced mitochondrial superoxide anion production and reduced cellular oxygen consumption.
