# Supplementary figures and images for: The Novel Elemene Derivative, OMe-Ph-Elemene, Attenuates Oxidative Phosphorylation and Facilitates Apoptosis by Inducing Intracellular Reactive Oxygen Species
Source: Antioxidants (Basel). 2024 Dec 9;13(12):1499. doi: 10.3390/antiox13121499 (PMC11672920; doi:10.3390/antiox13121499)

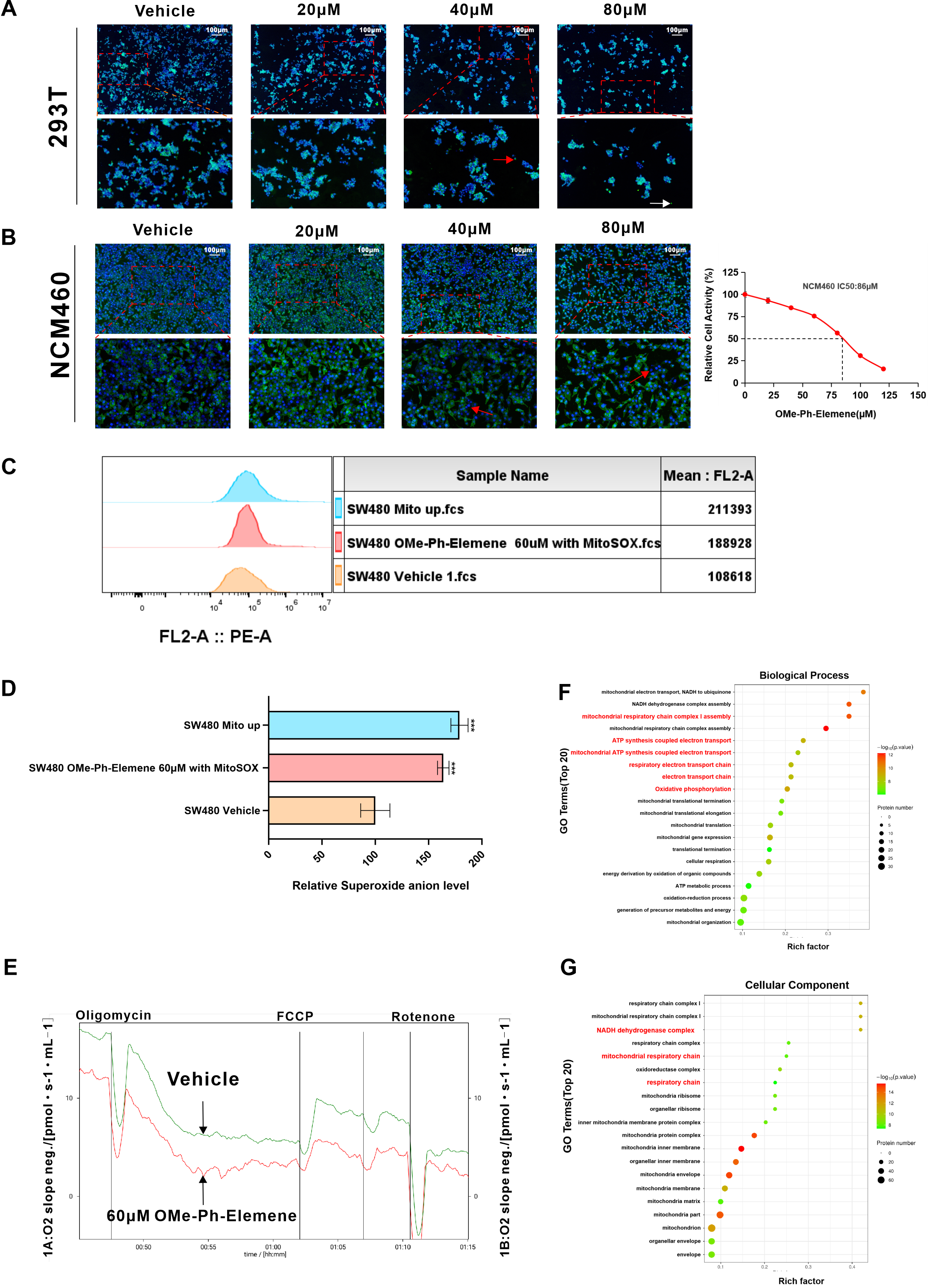

Supplement: Supplementary file 1 [file antioxidants-13-01499-s001.zip › antioxidants-3224808-supplementary.tif]
